# Research methodology used in the 50 most cited articles in the field of pediatrics: types of studies that become citation classics

**DOI:** 10.1186/s12874-020-00940-0

**Published:** 2020-03-17

**Authors:** Antonia Jelicic Kadic, Tanja Kovacevic, Edita Runjic, Ana Simicic Majce, Josko Markic, Branka Polic, Julije Mestrovic, Livia Puljak

**Affiliations:** 1 Department of Pediatrics, Split University Hospital, Split, Croatia; 2 Department of Pediatrics, General Hospital Dubrovnik, Dubrovnik, Croatia; 3 University of Split School of Medicine, Split, Croatia; 4 Center for Evidence-Based Medicine and Health Care, Catholic University of Croatia, Ilica 242, 10000 Zagreb, Croatia

**Keywords:** Study design, Citation density, Methodology, Pediatrics, Citation classics

## Abstract

**Background:**

One of the frequently used methods for assessing research trends and the impact of published scientific literature in a particular discipline is citation analysis. Journals may strive to improve their metrics by choosing manuscripts and study designs that are more likely to be cited. The aim of this study was to identify the 50 most-cited articles in the field of pediatrics, analyze their study design and other characteristics of those articles, and assess the prevalence of systematic reviews among them.

**Methods:**

In December 2017, we searched Web of Science (WoS) for all articles published in the field of pediatrics. Two authors screened articles independently and in the further analysis included 50 articles with the highest number of citations. To avoid bias for scientific papers published earlier, the citation density was calculated. We also analyzed Journal Impact Factor (JIF) of journals where citation classics were published.

**Results:**

The citation density in top 50 cited articles in the field of pediatrics ranged from 33.16 to 432.8, with the average of 119.95. Most of the articles reported clinical science. Median 2016 JIF for journals that published them was 6.226 (range: 2.778 to 72.406). Half of the top 10 highly cited articles in pediatrics were published in a journal with JIF below 5. Most of the studies among the citation classics in pediatrics were cross-sectional studies (*N* = 22), followed by non-systematic narrative reviews (*N* = 10), randomized controlled trials (*N* = 5), cohort studies (N = 5), systematic reviews (N = 2), case-control studies (N = 2), case reports (N = 2), and there was one study protocol and one expert opinion.

**Conclusion:**

Few randomized controlled trials and systematic reviews were among citation classics in the field of pediatrics. Articles that use observational research methodology, and are published in journals with lower impact factors, can become citation classics.

## Background

The growth of a particular scientific field can be gauged by research advancements that have been reported over time. One of the frequently used methods for assessing research trends and impact of published scientific literature in a particular discipline is citation analysis. By citing an article, other researchers in the field give acknowledgement and prominence to someone’s work, highlighting its importance for further advancement of knowledge [1].

Citations are also used to calculate metrics such as Journal Impact Factor (JIF) [2]. Editors of scholarly journals may seek manuscripts with a potential for attracting high citation counts, in an effort to increase their JIF, improve their status, and attract the best manuscripts in future [3–6]. Some manuscripts may receive disproportionate attention of other researchers and very high number of citations [7, 8]. Journal editors would thus benefit from knowing which manuscripts may attract higher citation counts, contribute towards JIF of their journal, and potentially become a citation classic.

Bibliometric analyses of the most-cited articles in certain disciplines, also called “citation classics”, provide insight into research areas that have marked certain discipline. The number of citations and citation density, i.e. average number of citations since the date a manuscript was published, can be used to indicate influence of a certain publication on a respective field [1].

It has been reported that systematic reviews and meta-analyses have a higher citation rate compared to other study designs [9–15]. However, it is unclear whether systematic reviews are predominant research methodology among the highest-cited articles in a certain field, i.e. ‘citation classics’.

The aim of this study was to identify the 50 most-cited articles in the field of pediatrics, to analyze their characteristics and to determine research methodology used in those articles.

## Methods

### Study design

This was a bibliometric study of articles published in scholarly journals.

### Study eligibility

We included in the analysis articles from the field of pediatrics published in scholarly journals indexed in Web of Science (WoS), regardless of the date and language of publication.

### Search

In December 2017, we searched the WoS database [Science Citation Index – Expanded (SCI-E), Social Sciences Citation Indeks (SSCI) and Art & Humanities with the Advanced Search] for all articles published in the field of pediatrics. The following search terms were used: pediatric* or paediatric* or child* or newborn* or neonat* or adolescen* or infan* or preschool* or pre-school* or teen* or kindergarden* or elementary school* or nursery school* or youth* or baby* or babies* or schoolchild* or toddler*.

When designing the search strategy, we used results of Kastner et al., who explored age-specific search strategies for MEDLINE [16], and reported that [quote]: “*The three-term strategy “adolescent.tw. OR children.tw. OR child, preschool.sh.“ yielded the best optimization of sensitivity and specificity (89.3% and 87.3%, respectively)*.” To increase the possibility to find all pediatric-related studies, we expanded on this search words, and used more labor-intensive approach – we have increased the number of search-terms related to children, to make sure that we find as many records as possible. We did not use any time or language limits.

### Screening and study selection

After conducting the search, the articles were sorted in WoS by highest citation number and exported. Two authors independently screened the top-ranking articles until 50 most cited articles in the field of pediatrics were identified. We excluded all articles that used the above mentioned keywords but that were not focused on pediatric population.

### Data extraction

Two authors independently categorized articles’ research methodology (i.e. study design). The studies were also categorized into the following categories: i) basic science, ii) clinical science, iii) reviews and guidelines and iv) diagnostic studies, and depending on pediatric subspecialty.

Additionally, two authors independently extracted the following data from each included article: number of authors, title, year of publication, journal name, 2016 JIF, country in which the study was performed, WoS category and WoS research area, number of citations and funding source.

To avoid bias towards older articles, which tend to have more citations because there has been more time to cite them, we calculated citation density for every study ranked in top 50 by dividing number of citations over the number of years the work had been published. Each article was assigned to a single country in accordance with the corresponding author’s address.

### Ethics

This study included analysis of published articles, and thus approval of a research ethics committee was not applicable.

### Statistical analysis

Kolmogorov-Smirnov and Shapiro-Wilk tests were used to test normality of data distribution. Spearman’s r was used to test correlations between variables when both variables were continuous. The Mood’s median test was used to test the equality of medians. Descriptive statistics and frequencies were calculated. Both descriptive and inferential statistics were used to present the results. Statistical significance was set at p<0.05. Statistical analysis was conducted using SPSS statistical software (SPSS 24.0, IBM® SPSS®, IBM Corporation, Armonk, NY, USA).

## Results

Number of records after the search was 3,204,914. We downloaded 500 with highest citation number and screened them. Table 1 presents a list of the 50 highest-cited articles in the field of pediatrics.

**Table 1:** The 50 highest-cited articles in the field of pediatrics, their first author, journal, citation density, number of citations and study design

Rank	First author	Journal	Journal Impact Factor	Citation density	Number of citations	Study design
1	Ogden CL. 2012 [17]	JAMA	44.405	432.8	2164	cross-sectional
2	Ogden CL. 2010 [18]	JAMA	44.405	264.1	1849	cross-sectional
3	Goodman R. 1997 [19]	Journal of Child Psychology and Psychiatry and Allied Disciplines	6.226	220.2	4403	cross-sectional
4	Kaufman J. 1997 [20]	Journal of the American Academy of Child and Adolescent Psychiatry	6.442	216.9	4338	case control study
5	Black RE. 2008 [21]	Lancet	47.831	215.9	1943	literature review
6	Polanczyk G. 2007 [22]	American Journal of Psychiatry	14.176	195.3	1953	systematic review
7	Barlow SE. 2007 [23]	Pediatrics	5.705	190.1	1901	expert opinion and literature review
8	De Onis M. 2007 [24]	Bulletin of the World Health Organization	4.939	184.9	1849	cross-sectional
9	Asher MI. 1995 [25]	Lancet	47.831	184.7	2032	cross-sectional
10	Felitti VJ. 1998 [26]	American Journal of Preventive Medicine	4.212	165.9	3152	cross-sectional
11	Spear LP. 2000 [27]	Neuroscience and Biobehavioral Reviews	8.299	161.9	2752	literature review
12	Strong WB. 2005 [28]	Journal of Pediatrics	3.874	155.9	1871	systematic review
13	Giedd JN. 1999 [29]	Nature Neuroscience	17.830	145.6	2621	cross-sectional
14	Palisano R. 1997 [30]	Developmental Medicine and Child Neurology	3.116	138.3	2765	cross-sectional and literature review
15	Filmer D. 2001 [31]	Demography	2.802	134.8	2157	cross-sectional
16	Hoffman JIE. 2002 [32]	Journal of the American College of Cardiology	19.896	134.5	2018	expert opinion and literature review
17	Sallis JF. 2000 [33]	Medicine and Science in Sports and Exercise	4.141	125.9	2140	literature review
18	Costello EJ. 2003 [34]	Archives of General Psychiatry	14.48 (2014)	124.5	1743	longitudinal cohort study
19	Masten AS. 2001 [35]	American Psychologist	6.681	121.8	1948	expert opinion and literature review
20	Beasley R. 1998 [36]	Lancet	47.831	119.5	2271	cross-sectional
21	Resnick MD. 1997 [37]	JAMA	44.405	115.3	2305	cross-sectional
22	Connor EM. 1994 [38]	New England Journal of Medicine	72.406	115.1	2648	randomized controlled trial
23	Varni JW. 2001 [39]	Medical Care	2.897	113.1	1809	cross-sectional
24	Martinez FD. 1995 [40]	New England Journal of Medicine	72.406	111.4	2451	expert opinion and literature review
25	Shaffer D. 2000 [41]	Journal of the American Academy of Child and Adolescent Psychiatry	6.442	111.4	1894	prospective cohort study
26	Brenner DJ. 2000 [42]	American Journal of Roentgenology	2.778	102.9	1749	expert opinion and literature review
27	Montague CT. 1997 [43]	Nature	40.137	96.8	1935	case report (two cases)
28	Saffran JR. 1996 [44]	Science.	37.205	96.2	2012	cross-sectional
29	Crick NR. 1995 [45]	Psychological Bulletin	16.793	96.0	2207	literature review
30	Jensen PS. 1999 [46]	Archives of General Psychiatry	14.48 (2014)	95.7	1722	randomized controlled trial
31	Baroncohen S. 1985 [47]	Cognition	3.414	91.9	2941	case control study
32	Asher MI. 1995 [48]	European Respiratory Journal	10.569	91.1	2941	study protocol
33	Ames C. 1992 [49]	Journal of Educational Psychology	3.459	89.6	2240	expert opinion (descriptive, qualitative)
34	Holmbeck GN. 1997 [50]	Journal of Consulting and Clinical Psychology.	4.593	86.1	1722	expert opinion and literature review
35	Papile LA. 1978 [51]	Journal of Pediatrics	3.874	84.0	3277	cross-sectional
36	Czeizel AE. 1992 [52]	New England Journal of Medicine	72.406	82.9	2073	randomized controlled trial
37	Crick NR. 1995 [53]	Child Development	4.195	82.3	1811	cross-sectional
38	Miller NJ. 1993 [54]	Clinical Science	4.936	80.1	1923	cross-sectional
39	Wald N. 1991 [55]	Lancet	47.831	77.8	2023	randomized controlled trial
40	Wimmer H. 1983 [56]	Cognition	3.414	72.6	2470	cross-sectional
41	Barker DJP. 1989 [57]	Lancet	47.831	65.0	1819	retrospective cohort study
42	Harter S. 1982 [58]	Child Development	4.195	59.0	2065	cross-sectional
43	Mulliken JB. 1982 [59]	Plastic and Reconstructive Surgery	3.843	54.6	1910	cross-sectional
44	Shaffer D. 1983 [60]	Archives of General Psychiatry	14.48 (za 2014)	53.5	1818	cross-sectional
45	Dubowitz LM. 1970 [61]	Journal of Pediatrics	3.874	48.0	2255	cross-sectional
46	Jones KL. 1973 [62]	Lancet	47.831	42.0	1849	case report (two cases)
47	Schwartz GJ. 1976 [63]	Pediatrics	5.705	41.7	1708	cross-sectional
48	Liggins GC. 1972 [64]	Pediatrics	5.705	41.2	1852	randomized controlled trial
49	Tanner JM. 1966 [65]	Archives of Disease in Childhood.	3.265	33.6	1714	longitudinal cohort study
50	Tanner JM. 1966 [66]	Archives of Disease in Childhood.	3.265	33.2	1691	longitudinal cohort study

### Research methodology used in pediatrics’ citation classics

Most of the studies among the citation classics in pediatrics were cross-sectional studies (*N* = 22), followed by non-systematic narrative reviews (*N* = 10), randomized controlled trials (*N* = 5), cohort studies (N = 5), systematic reviews (N = 2), case-control studies (N = 2), case reports (N = 2), and there was one study protocol and one expert opinion.

Among the only two systematic reviews among the pediatrics’ citation classics, neither one was a Cochrane review. One was published in the American Journal of Psychiatry in 2007, and it addressed world-wide prevalence of Attention-Deficit/Hyperactivity Disorder (ADHD) [22], while the other one was published in Journal of Pediatrics in 2005 on the subject of physical activity for school-aged youth [28].

### Characteristics of pediatrics’ citation classics

**Fig. 1:**
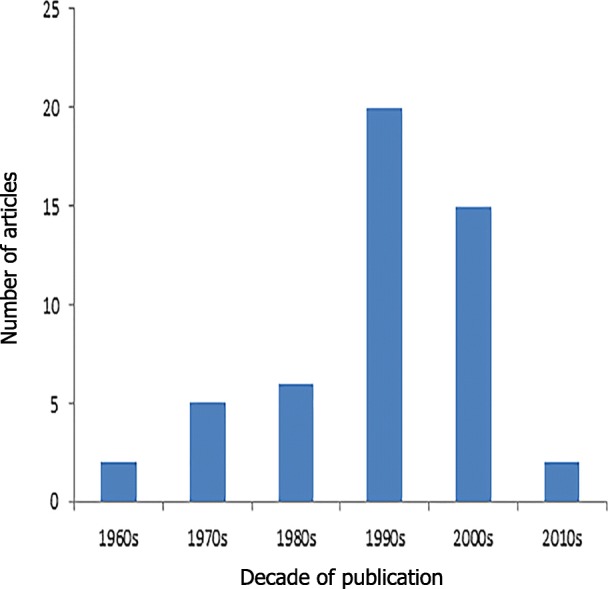
Number of top 50 cited articles by decade of publication

**Fig. 2:**
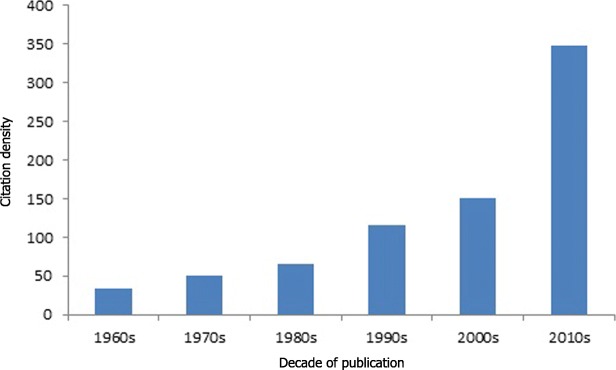
Citation density of articles in top 50 by decade of publication

The top 50 cited articles in pediatrics were published from 1966 to 2012. The majority of the studies were published before year 2000 (33/50; 66%); analysis per decade indicates that the highest number of those articles were published in 1990s (*N* = 20) (Fig. 1). Mean value for years passed since publication was 23.24 (range: 5 to 51 years) and 1st quartile value is Q1 = 16. Citation density increased with increasing decade (Fig. 2) and there was a high, statistically significant positive correlation between citation density and year of publication (Spearman’s *ρ* = 0.888, *p* < 0.001).

The citation density in top 50 cited articles in the field of pediatrics ranged from 33.16 to 432.8, with the average of 119.95. The analysis showed an existing, but low positive correlation between citation density and the number of citations (Spearman’s *ρ* = 0.369; *p* = 0.008). Article with highest density was published by Ogden et al. in JAMA, 2012; the article reported data about obesity and body mass index in US children and adolescents in 1990–2010 [17].

The article with highest number of citations was published by Goodman et al. in 1997; the article was cited total of 4403 times, it covered pediatric psychology, namely strengths and difficulties questionnaire [19].

The oldest articles in top 50 were two articles by Tanner et al. about standards from birth to maturity for height weight height velocity and weight velocity, which was published in 1966 [65, 66]. Both were published in *Archives of disease in childhood* with impact factor 3.265 in 2016.

Of the top 50 ranked articles, 33 corresponding author’s address were affiliated with institutions based in the United States. Other affiliations were from United Kingdom, New Zealand, Austria, Hungary, Sweden and Brazil. Most of them had 5 or more authors (*N* = 24). Information about funding was reported in 37 articles; studies were funded mostly by non-profit grants.

The majority of the top 50 studies in pediatrics reported topics about child psychology (*N* = 13) and psychiatry (*N* = 8). Others reported findings from the sub-specialties of neonatology (*N* = 7), epidemiology (*N* = 4), allergology (*N* = 3), general pediatrics (N = 3), growth and development (N = 3) cardiology (N = 2), endocrinology (N = 3), genetics (N = 1), nephrology (N = 1), neuropediatrics (N = 1) and biochemistry (N = 1).

Only one study was categorized as basic science, 28 were clinical studies, 12 were reviews or guidelines and 9 were diagnostic. We did not find statistically significant correlations found between citation density and category of studies (*ρ* = 0.709; *p* = 0.054). Also, Mood’s median test did not suggest statistically significant differences in citation density across different categories (*p* = 0.407).

The top 50 cited articles in the field of pediatrics were published in 25 different journals. Most of them were published in *The Lancet*, a general medical journal (*N* = 6). Other journals that published more than one top 50 pediatric article were: *Archives of General Psychiatry* (*N* = 3), *JAMA* (N = 3), *Journal of Pediatrics* (N = 3), *New England Journal of Medicine* (N = 3), *Pediatrics* (N = 3), *Archives of Disease in Childhood* (*N* = 2), *Child Development* (N = 2), *Cognition* (N = 2) and *Journal of the American Academy of Child and Adolescent Psychiatry* (N = 2).

The 2016 impact factor for journals that published the top 50 cited pediatric articles ranged from 2.778 to 72.406 with median of 6.226. Half of the top 10 highly cited articles in pediatrics were published in a journal with impact factor below 5.

All data collected within this study, for all analyzed variables, are available in Supplementary file 1.

## Discussion

The majority of pediatrics’ citation classics were observational studies with cross-sectional study design, and half of them were published in a journal with JIF below 5. Pediatrics’ citation classics were published from 1966 to 2012, with the majority published in 1990s. Most of the articles had 5 or more authors and were funded, mostly from non-profit grants. The majority of the top 50 studies in pediatrics reported topics about child psychology and psychiatry.

While it has been reported earlier that evidence syntheses, such as systematic reviews and meta-analyses, receive a higher citation rate compared to other study designs [9–15], our study indicates that there were very few systematic reviews among citation classics in the specialized field of pediatrics. This indicates that editors need not favor those kinds of study designs, when making decisions about which manuscripts to accept and publish.

We found a significant correlation between citation density and year of publication were citation density increased with increasing decade. Similar results found Baldwin et al. [67] who analyzed the highest-cited articles in pediatric orthopedic surgery.

In this study we found that the top 50 cited articles in the field of pediatrics were published in a wide range of journals according to the 2016 impact factor. Almost half of the articles were published in a journal with impact factor above 20, but the journal impact factors ranged from 2.778 to 72.406. It is encouraging to researchers to see that even studies published in journals with modest impact factors can become highly cited. It is particularly encouraging to see that half of the top 10 highly cited articles in pediatrics were published in a journal with impact factor below 5. These figures indicate a wide range of citation patterns between individual journals, suggesting that impact factor of journal should not be considered as an essential feature for article’s number of citations and that even a manuscript published in a lower-ranked journal can become a citation classic.

In 1977, Eugene Garfield described a concept of a “citation classic” in commentaries that were published in the Current Contents about the most-cited papers ever published [68]. Although number of citations varies in different disciplines, in general, it was postulated that a publication cited more than 400 times should be considered a citation classic, with the acknowledgement that in some fields, where there are fewer researchers, even 100 citations may qualify article as a citation classic [69].

In this study we restricted our analysis to the top 50 highly cited articles in the field of pediatrics, and all of these articles had much more than 400 citations, ranging from 4403 for the top-ranking article to 1691 for the 50th article on the list. This is understandable as we chose very broad field of pediatrics for this particular bibliometric analysis. Pediatrics encompasses various medical sub-specialties, which is reflected in the various topics that we can find among the top 50 articles we identified in this study.

Two article with the highest citation density reported trends in obesity and BMI index in US children and adolescents. Prevalence of childhood obesity has reached epidemic pro. The cost of obesity in childhood and adolescents for society is significant and quantifiable [70].

It has been suggested that childhood obesity is a risk factor for select cardiovascular diseases in adulthood [71], which is worrying because CVD are the predominant cause of death. It has been shown that obesity, glucose intolerance, and hypertension in childhood population were strongly associated with increased rates of premature death from endogenous causes [72].

### Limitations

A limitation of this study might be approach to choosing the top 50 articles using the absolute 2016 impact factor. We first extracted highest-cited articles, and then for each of the top 50 cited articles in pediatrics we analyzed a citation density, defined as number of citations per year. Even though we used this method to avoid bias, it is possible that there are pediatric articles with a higher citation density that are not included in our top 50 list.

Regarding the citation windows, there are no uniform recommendations. It has been reported that there are significant variations in citation ageing between different research fields, types of articles, total citation counts and months of publication. The within-group differences were also shown to be even more striking, as many articles in the slowest ageing field might still age faster than many articles in the fastest ageing field. Furthermore, it has been observed that field normalization cannot impact the accuracy of using short citation time windows [73].

Also, there may be factors, such as document type, which maybe correlated with number of citations [7, 8, 74, 75], but comprehensive analysis of such factors on a large sample of our studies was not the aim of our study. Furthermore, we did not use any method of normalization, which may have added to the results.

Furthermore, field of pediatrics is very broad, and the articles included come from various subfields of pediatrics. However, any article, regardless of its subfield, has a potential to become a citation classic. This is also reflected in the variety of topics covered in our top 50 articles.

In this study we focused on identifying the 50 most-cited articles, while other approaches could be also used, such as identifying the top 5 or 10% of the highest-cited articles in the field. However, in bibliometric studies, there is no uniform recommendation regarding study design of these types of bibliometric studies. For example, it has been reported that in the biomedical field, list of fifty or more citations is common, while in mathematics less than twenty citations are most common [76].

Future studies in this field could address some of these limitations.

## Conclusion

Few randomized controlled trials and systematic reviews were among citation classics in the field of pediatrics. Articles that use observational research methodology, and are published in journals with lower impact factors, can become citation classics. This implies that journal editors should not have a preferential study design when making decisions what to accept and publish, and instead to focus on the quality and contribution of a manuscript, regardless of the study design.

## Supplementary information

**Additional file 1.** Supplementary file 1. Data for all characteristics of pediatrics’ citation classics that were analyzed within the study. The Excel table contains Supplementary file 1, with all data about characteristics of the 50 highest-cited articles in the field of pediatrics. The Excel table contains the following columns: name of the first author, country of authors’ affiliation, year of publication, years passed since publication, title, times cited in all databases, citation density, Journal Impact Factor for year 2016, Web of Science category, research area, funding source, study design, number of authors.

## Data Availability

All data generated within this study are available in the Supplementary file 1.

## Abbreviations

ADHD: Attention-Deficit/Hyperactivity Disorder
JIF: Journal Impact Factor
WoS: Web of Science

## References

[1] Malhotra Konark, Saeed Omar, Goyal Nitin, Katsanos Aristeidis H., Tsivgoulis Georgios (2018). Top-100 Highest-Cited Original Articles in Ischemic Stroke: A Bibliometric Analysis. World Neurosurgery.

[2] Opthof T (1997). Sense and nonsense about the impact factor. Cardiovasc Res.

[3] Jawaid SA, Jawaid M (2018). Impact factor is off the ventilator: survives and is thriving. Pak J Med Sci.

[4] Arguelles JC, Arguelles-Prieto R. Are the Editors Responsible for Our Obsession with the Impact Factor? mBio. 2017;8(6).e02019–17. 10.1128/mBio.02019-17 PMC5736912 29259087

[5] Watson R, Cleary M, Hunt GE (2013). What gets highly cited in JAN? Can editors pick which articles will contribute to a journal's impact factor?. J Adv Nurs.

[6] Falagas ME, Alexiou VG (2008). The top-ten in journal impact factor manipulation. Arch Immunol Ther Exp.

[7] Timsit JF, Citerio G, Lavilloniere M, Perner A, Ruckly S, Bakker J, Bassetti M, Benoit D, Curtis JR, Doig GS (2019). Determinants of downloads and citations for articles published in intensive care medicine. Intensive Care Med.

[8] Murray SB, Pila E, Mond JM, Mitchison D, Nauman E, Griffiths S (2018). Global trends in high impact psychiatry research. World Psychiatry.

[9] Allareddy V, Lee MK, Shah A, Elangovan S, Lin CY (2012). Association between study design and citation counts of articles published in the American journal of orthodontics and Dentofacial orthopedics and angle orthodontist. Orthodontics.

[10] Bhandari M, Busse J, Devereaux PJ, Montori VM, Swiontkowski M, Tornetta Iii P, Einhorn TA, Khera V, Schemitsch EH (2007). Factors associated with citation rates in the orthopedic literature. Can J Surg.

[11] Montori VM, Wilczynski NL, Morgan D, Haynes RB (2003). Systematic reviews: a cross-sectional study of location and citation counts. BMC Med.

[12] Okike K, Kocher MS, Torpey JL, Nwachukwu BU, Mehlman CT, Bhandari M (2011). Level of evidence and conflict of interest disclosure associated with higher citation rates in orthopedics. J Clin Epidemiol.

[13] Patsopoulos NA, Analatos AA, Ioannidis JP (2005). Relative citation impact of various study designs in the health sciences. Jama.

[14] Willis DL, Bahler CD, Neuberger MM, Dahm P (2011). Predictors of citations in the urological literature. BJU Int.

[15] Winnik S, Raptis DA, Walker JH, Hasun M, Speer T, Clavien PA, Komajda M, Bax JJ, Tendera M, Fox K (2012). From abstract to impact in cardiovascular research: factors predicting publication and citation. Eur Heart J.

[16] Kastner M, Wilczynski NL, Walker-Dilks C, McKibbon KA, Haynes B (2006). Age-specific search strategies for Medline. J Med Internet Res.

[17] Ogden CL, Carroll MD, Kit BK, Flegal KM (2012). Prevalence of obesity and trends in body mass index among US children and adolescents, 1999-2010. JAMA.

[18] Ogden CL, Carroll MD, Curtin LR, Lamb MM, Flegal KM (2010). Prevalence of high body mass index in US children and adolescents, 2007-2008. JAMA.

[19] Goodman R (1997). The strengths and difficulties questionnaire: a research note. J Child Psychol Psychiatry Allied Disciplines.

[20] Kaufman J, Birmaher B, Brent D, Rao U, Flynn C, Moreci P, Williamson D, Ryan N (1997). Schedule for affective disorders and schizophrenia for school-age children present and lifetime version (K-SADS-PL): initial reliability and validity data. J Am Acad Child Adolesc Psychiatry.

[21] Black RE, Allen LH, Bhutta ZA, Caulfield LE, de Onis M, Ezzati M, Mathers C, Rivera J (2008). Maternal, child Undernutr S: maternal and child undernutrition 1 - maternal and child undernutrition: global and regional exposures and health consequences. Lancet.

[22] Polanczyk G, de Lima MS, Horta BL, Biederman J, Rohde LA (2007). The worldwide prevalence of ADHD: a systematic review and metaregression analysis. Am J Psychiatr.

[23] Barlow SE (2007). Expert committee recommendations regarding the prevention, assessment, and treatment of child and adolescent overweight and obesity: summary report. Pediatrics.

[24] de Onis M, Onyango AW, Borghi E, Siyam A, Nishida C, Siekmann J (2007). Development of a WHO growth reference for school-aged children and adolescents. Bull World Health Organ.

[25] Asher MI, Montefort S, Bjorksten B, Lai CKW, Strachan DP, Weiland SK, Williams H, Grp IPTS (2006). Worldwide time trends in the prevalence of symptoms of asthma, allergic rhinoconjunctivitis, and eczema in childhood: ISAAC phases one and three repeat multicountry cross-sectional surveys. Lancet.

[26] Felitti VJ, Anda RF, Nordenberg D, Williamson DF, Spitz AM, Edwards V, Koss MP, Marks JS (1998). Relationship of childhood abuse and household dysfunction to many of the leading causes of death in adults - the adverse childhood experiences (ACE) study. Am J Prev Med.

[27] Spear LP (2000). The adolescent brain and age-related behavioral manifestations. Neurosci Biobehav Rev.

[28] Strong WB, Malina RM, Blimkie CJR, Daniels SR, Dishman RK, Gutin B, Hergenroeder AC, Must A, Nixon PA, Pivarnik JM (2005). Evidence based physical activity for school-age youth. J Pediatr.

[29] Giedd JN, Blumenthal J, Jeffries NO, Castellanos FX, Liu H, Zijdenbos A, Paus T, Evans AC, Rapoport JL (1999). Brain development during childhood and adolescence: a longitudinal MRI study. Nat Neurosci.

[30] Palisano R, Rosenbaum P, Walter S, Russell D, Wood E, Galuppi B (1997). Development and reliability of a system to classify gross motor function in children with cerebral palsy. Dev Med Child Neurol.

[31] Filmer D, Pritchett LH (2001). Estimating wealth effects without expenditure data - or tears: an application to educational enrollments in states of India. Demography.

[32] Hoffman JIE, Kaplan S (2002). The incidence of congenital heart disease. J Am Coll Cardiol.

[33] Sallis JF, Prochaska JJ, Taylor WC (2000). A review of correlates of physical activity of children and adolescents. Med Sci Sports Exerc.

[34] Costello EJ, Mustillo S, Erkanli A, Keeler G, Angold A (2003). Prevalence and development of psychiatric disorders in childhood and adolescence. Arch Gen Psychiatry.

[35] Masten AS (2001). Ordinary magic - resilience processes in development. Am Psychol.

[36] Beasley R, Keil U, von Mutius E, Pearce N, Ait-Khaled N, Anabwani G, Anderson HR, Asher MI, Bjorkstein B, Burr ML (1998). Worldwide variation in prevalence of symptoms of asthma, allergic rhinoconjunctivitis, and atopic eczema: ISAAC. Lancet.

[37] Resnick MD, Bearman PS, Blum RW, Bauman KE, Harris KM, Jones J, Tabor J, Beuhring T, Sieving RE, Shew M (1997). Protecting adolescents from harm - findings from the National Longitudinal Study on adolescent health. JAMA.

[38] Connor EM, Sperling RS, Gelber R, Kiselev P, Scott G, Osullivan MJ, Vandyke R, Bey M, Shearer W, Jacobson RL (1994). Reduction of maternal-infant transmission of human-immunodeficiency-virus type-1 with zidovudine treatment. N Engl J Med.

[39] Varni JW, Seid M, Kurtin PS (2001). PedsQL (TM) 4.0: reliability and validity of the pediatric quality of life inventory (TM) version 4.0 generic core scales in healthy and patient populations. Med Care.

[40] Martinez FD, Wright AL, Taussig LM, Holberg CJ, Halonen M, Morgan WJ, Bean J, Bianchi H, Curtiss J, Ey J (1995). Asthma and wheezing in the first 6 years of life. N Engl J Med.

[41] Shaffer D, Fisher P, Lucas CP, Dulcan MK, Schwab-Stone ME (2000). NIMH diagnostic interview schedule for children version IV (NIMH DISC-IV): description, differences from previous versions, and reliability of some common diagnoses. J Am Acad Child Adolesc Psychiatry.

[42] Brenner DJ, Elliston CD, Hall EJ, Berdon WE (2001). Estimated risks of radiation-induced fatal cancer from pediatric CT. Am J Roentgenol.

[43] Montague CT, Farooqi IS, Whitehead JP, Soos MA, Rau H, Wareham NJ, Sewter CP, Digby JE, Mohammed SN, Hurst JA (1997). Congenital leptin deficiency is associated with severe early-onset obesity in humans. Nature.

[44] Saffran JR, Aslin RN, Newport EL (1996). Statistical learning by 8-month-old infants. Science.

[45] Crick NR, Dodge KA (1994). A review and reformulation of social information-processing mechanisms in childrens social-adjustment. Psychol Bull.

[46] Jensen PS, Arnold LE, Richters JE, Severe JB, Vereen D, Vitiello B, Schiller E, Hinshaw SP, Elliott GR, Conners CK (1999). A 14-month randomized clinical trial of treatment strategies for attention-deficit/hyperactivity disorder. Arch Gen Psychiatry.

[47] Baroncohen S, Leslie AM, Frith U (1985). Does the autistic-child have a theory of mind. Cognition.

[48] Asher MI, Keil U, Anderson HR, Beasley R, Crane J, Martinez F, Mitchell EA, Pearce N, Sibbald B, Stewart AW (1995). International study of asthma and allergies in childhood (ISAAC) - rationale and methods. Eur Respir J.

[49] Ames C (1992). Classrooms - goals, structures, and student motivation. J Educ Psychol.

[50] Holmbeck GN (1997). Toward terminological, conceptual, and statistical clarity in the study of mediators and moderators: examples from the child-clinical and pediatric psychology literatures. J Consult Clin Psychol.

[51] Papile LA, Burstein J, Burstein R, Koffler H (1978). Incidence and evolution of subependymal and intra-ventricular hemorrhage - study of infants with birth weights less than 1,500 gm. J Pediatr.

[52] Czeizel AE, Dudas I (1992). Prevention of the 1st occurrence of neural-tube defects by periconceptional vitamin supplementation. N Engl J Med.

[53] Crick NR, Grotpeter JK (1995). Relational aggression, gender, and social-psychological adjustment. Child Dev.

[54] Miller NJ, Riceevans C, Davies MJ, Gopinathan V, Milner A (1993). A novel method for measuring antioxidant capacity and its application to monitoring the antioxidant status in premature neonates. Clin Sci.

[55] Wald N (1991). Prevention of neural-tube defects - results of the medical-research-council vitamin study. Lancet.

[56] Wimmer H, Perner J (1983). Beliefs about beliefs - representation and constraining function of wrong beliefs in young childrens understanding of deception. Cognition.

[57] Barker DJP, Winter PD, Osmond C, Margetts B, Simmonds SJ (1989). Weight in infancy and death from ischemic heart-disease. Lancet.

[58] Harter S (1982). The perceived competence scale for children. Child Dev.

[59] Mulliken JB, Glowacki J (1982). Hemangiomas and vascular malformations in infants and children - a classification based on endothelial characteristics. Plast Reconstr Surg.

[60] Shaffer D, Gould MS, Brasic J, Ambrosini P, Fisher P, Bird H, Aluwahlia S (1983). A childrens global assessment scale (CGAS). Arch Gen Psychiatry.

[61] Dubowitz LM, Dubowitz V, Goldberg C (1970). Clinical assessment of gestational age in newborn infant. J Pediatr.

[62] Jones KL, Smith DW (1973). Recognition of fetal alcohol syndrome in early infancy. Lancet.

[63] Schwartz GJ, Haycock GB, Edelmann CM, Spitzer A (1976). Simple estimate of glomerular-filtration rate in children derived from body length and plasma creatinine. Pediatrics.

[64] Liggins GC, Howie RN (1972). Controlled trial of antepartum glucocorticoid treatment for prevention of respiratory distress syndrome in premature infants. Pediatrics.

[65] Tanner JM, Whitehouse RH, Takaishi M (1966). Standards From Birth To Maturity For Height Weight Height Velocity And Weight Velocity - British Children 1965 .I. Arch Dis Child.

[66] Tanner JM, Whitehouse RH, Takaishi M (1966). Standards From Birth To Maturity For Height Weight Height Velocity And Weight Velocity - British Children 1965 .2. Arch Dis Child.

[67] Baldwin KDKK, Namdari S, Sankar W, Flynn JM, Dormans JP (2012). The 50 most cited articles in pediatric orthopedic surgery. J Pediatr Orthop B.

[68] Garfield E (1977). Introducing citation classics: the human side of Sciencific reports. Essays Info Sci.

[69] Karnisova L, Hradsky O, Blahova K, Fencl F, Dolezel Z, Zaoral T, Zieg J (2018). Complement activation is associated with more severe course of diarrhea-associated hemolytic uremic syndrome, a preliminary study. Eur J Pediatr.

[70] Broyles ST, Denstel KD, Church TS, Chaput JP, Fogelholm M, Hu G, Kuriyan R, Kurpad A, Lambert EV, Maher C (2015). The epidemiological transition and the global childhood obesity epidemic. Int J Obes Suppl.

[71] Umer A, Kelley GA, Cottrell LE, Giacobbi P, Innes KE, Lilly CL (2017). Childhood obesity and adult cardiovascular disease risk factors: a systematic review with meta-analysis. BMC Public Health.

[72] Franks PW, Hanson RL, Knowler WC, Sievers ML, Bennett PH, Looker HC (2010). Childhood obesity, other cardiovascular risk factors, and premature death. N Engl J Med.

[73] Jian W (2012). Citation time window choice for research impact evaluation. Scientometrics.

[74] Antoniou GA, Antoniou SA, Georgakarakos EI, Sfyroeras GS, Georgiadis GS (2015). Bibliometric analysis of factors predicting increased citations in the vascular and endovascular literature. Ann Vasc Surg.

[75] Ruano-Ravina A, Alvarez-Dardet C (2012). Evidence-based editing: factors influencing the number of citations in a national journal. Ann Epidemiol.

[76] Dorta-Gonzáleza Pablo D-GMI, Santos-Peñate DR, Suárez-Vega R (2014). Journal topic citation potential and between-field comparisons: the topic normalized impact factor. J Inform.

